# Thyroid function after menopause: is there any concern in thyroidology?

**DOI:** 10.1590/1806-9282.7012EDI

**Published:** 2024-12-16

**Affiliations:** 1 Laboratório de Ginecologia Estrutural e Molecular (LIM-58), Disciplina de Ginecologia, Departamento de Obstetrícia e Ginecologia, Hospital das Clínicas, Faculdade de Medicina, Universidade de São Paulo – São Paulo (SP), Brazil.; 2 Giresun Gynaecology and Pediatrics Education and Research Hospital, Division of Perinatology – Giresun, Turkey.; 3 Giresun Gynaecology and Pediatrics Education and Research Hospital, Department of Gynecology and Obstetrics – Giresun, Turkey.; 4 Giresun University, Faculty of Medicine, Department of Pathology – Giresun, Turkey.; 5 Giresun University, Faculty of Medicine, Division of Endocrine Surgery – Giresun, Turkey.; 6 Giresun University, Faculty of Medicine, Department of General Surgery – Giresun, Turkey.

Menopause, per se, brings forth explicit physiological and biological alterations that affect assorted bodily systems, embodying the delicate endocrine gland, the thyroid. Throughout their lives, women experience symptoms related to hormonal fluctuations, such as headaches, muscle cramps, sleep disturbances, and mood swings. Thyroid dysfunction is another health concern that commonly arises during menopause. A clear link has been established between aging and thyroid dysfunction, with postmenopausal women being at increased risk due to age-related shifts in thyroid function^1^. Herein, this paper addresses the concerns surrounding thyroid function in postmenopausal women, including diagnostic challenges, the impact of aging on thyroid health, and associated risks such as osteoporosis and malignity in thyroidology.

As women age, their endocrine system, including the thyroid gland, undergoes significant changes even without apparent disease. This process leads to a decline in T4 (thyroxine) production and an attenuated thyroid response to thyroid-stimulating hormone (TSH), resulting in higher TSH levels^2–4^. TSH has been reported to follow a U-shaped curve in iodine-sufficient populations, with elevated levels in both younger and older individuals, while FT3 (free triiodothyronine) levels diminish with age^5,6^. This de-escalation in thyroid function may provide a survival advantage for older adults, while younger individuals with low gland activity are at higher risk for cardiovascular diseases^7^. Therefore, age-specific reference intervals for thyroid evaluation in older women are crucial. In iodine-sufficient areas, older women are more likely to develop hypothyroidism^8^, a condition that can mimic many menopausal symptoms, including fatigue, memory problems, and muscle cramps^9,10^. Similarly, hyperthyroidism can manifest with symptoms such as anxiety, sweating, palpitations, and insomnia symptoms also common during menopause, making diagnosis difficult^11^. Thyroid hormones play an essential role in brain function by regulating cellular metabolism, gene expression, and neurotransmitter production, much like estrogen^12^. As estrogen levels decline during menopause, it becomes increasingly important to distinguish between menopausal symptoms and thyroid dysfunction since both can impact cognitive health^13^.

Diagnosing thyroid dysfunction in postmenopausal women is often complicated by the overlap of symptoms between menopause and thyroid disorders. Both conditions can cause similar symptoms, such as palpitations, insomnia, and weight gain, making differentiation difficult. Additionally, interpreting thyroid function test results can be challenging due to age, comorbidities, and ongoing treatments^7^. To address this, the American Society of Clinical Endocrinology recommends routine thyroid screening for older women, especially those undergoing menopause^14^. Postmenopausal women face an increased risk of osteoporosis and fractures, conditions that can be worsened by thyroid dysfunction. Hyperthyroidism, including its subclinical forms, is associated with lower bone mineral density (BMD) and an elevated risk of fractures, particularly in the spine^15^. Several factors, including declining metabolic clearance and medication interactions, affect thyroxine requirements in the elderly. Excessive doses of thyroxine may lead to severe issues such as atrial fibrillation, bone density loss, and other tachyarrhythmias. As such, careful thyroid function monitoring and personalized treatment are essential to maintaining bone health in postmenopausal women^16^. In cases where TSH suppression is required after thyroidectomy for thyroid carcinoma, stringent suppression might negatively impact BMD and raise the risk of fractures, highlighting the importance of balancing the benefits of TSH suppression with its potential impact on bone health^15,16^. Moreover, thyroid dysfunction is also linked to sexual dysfunction in women, a problem often exacerbated during menopause. While some studies suggest that correcting thyroid dysfunction can improve sexual function, the relationship between thyroid hormone levels and sexual health remains unclear. Of note, autoimmune thyroid diseases, in particular, have been associated with sexual dysfunction in women. Recently, an association between thyroid dysfunction and breast cancer risk, influenced by menopausal status, has been announced. As such, hyperthyroidism appears to increase the risk of breast cancer in postmenopausals, while hypothyroidism shows a stronger inverse relationship in premenopausal women. Thyroid hormones may contribute to breast cancer development through estrogen-like pathways. Additionally, an elevated thyroid hormone-to-estradiol ratio could promote breast cancer in postmenopausal women^17,18^. Of note, these findings might help to identify at-risk women and improve understanding of the hormonal mechanisms involved in breast cancer^19^.

Given the symptom overlap between thyroid dysfunction and menopause, healthcare providers should remain vigilant in detecting thyroid disorders in postmenopausal women. In thyroidology, routine screening for thyroid disease^20–25^ is recommended, particularly for women with a history of surgical menopause or other risk factors. To this end, thyroidologists should also be mindful of potential issues with thyroid function tests, such as the interference caused by biotin supplements^26,27^. A personalized approach to treatment is crucial when addressing both menopausal symptoms and thyroid dysfunction. Menopausal hormone therapy (MHT) can be a safe and effective option for managing both conditions, with evidence showing that MHT can prevent hip fractures without increasing thyroid cancer risk. However, it is essential, in order to avoid both under-treatment and over-treatment of thyroid disorders to reduce risks to cardiovascular and bone health^11^.

Thyroid dysfunction poses significant challenges for postmenopausal women, as it can worsen or mimic menopausal symptoms, complicating diagnosis. Age-related changes in thyroid function, the augmented risk of thyroid carcinoma, and the impact of thyroid disorders on bone health highlight the need for routine screening and personalized care. Healthcare providers should take a patient-centered approach to be able to ensure the best outcomes for postmenopausals, considering their unique health requirements and preferences.

## References

[1] Soares JM, Detanac D, Sengul I, Dugalic S, Sengul D, Detanac D (2024). Melatonin, menopause, and thyroid function in gynecologic endocrinology: what is the role?. Rev Assoc Med Bras (1992).

[2] Mintziori G, Veneti S, Poppe K, Goulis DG, Armeni E, Erel CT (2024). EMAS position statement: thyroid disease and menopause. Maturitas.

[3] Taylor PN, Lansdown A, Witczak J, Khan R, Rees A, Dayan CM (2023). Age-related variation in thyroid function - a narrative review highlighting important implications for research and clinical practice. Thyroid Res.

[4] Sengul D, Sengul I, Soares JM (2022). Repercussion of thyroid dysfunctions in thyroidology on the reproductive system: conditio sine qua non?. Rev Assoc Med Bras (1992).

[5] Noth RH, Mazzaferri EL (1985). Age and the endocrine system. Clin Geriatr Med.

[6] Franceschi C, Ostan R, Mariotti S, Monti D, Vitale G (2019). The aging thyroid: a reappraisal within the geroscience ıntegrated perspective. Endocr Rev.

[7] Kolanu BR, Vadakedath S, Boddula V, Kandi V (2020). Activities of serum magnesium and thyroid hormones in pre-, peri-, and post-menopausal women. Cureus.

[8] Sengul D, Sengul I (2018). Is there any link between a kind of thyrocyte dysfunction, hypothyroidism, and inflammatory hematologic parameters in the cases having the benign thyroid nodules?: a 5-year single-centre experience. Sanamed.

[9] Veggel KM, Ivarson DM, Rondeel JMM, Mijnhout GS (2022). Iodine deficiency in patients with hypothyroidism: a pilot study. J Thyroid Res.

[10] Gietka-Czernel M (2017). The thyroid gland in postmenopausal women: physiology and diseases. Prz Menopauzalny.

[11] Slopien R, Owecki M, Slopien A, Bala G, Meczekalski B (2020). Climacteric symptoms are related to thyroid status in euthyroid menopausal women. J Endocrinol Invest.

[12] Richard S, Ren J, Flamant F (2023). Thyroid hormone action during GABAergic neuron maturation: the quest for mechanisms. Front Endocrinol (Lausanne).

[13] Russell JK, Jones CK, Newhouse PA (2019). The role of estrogen in brain and cognitive aging. Neurotherapeutics.

[14] Garber JR, Cobin RH, Gharib H, Hennessey JV, Klein I, Mechanick JI (2012). Clinical practice guidelines for hypothyroidism in adults: cosponsored by the American Association of Clinical Endocrinologists and the American Thyroid Association. Thyroid.

[15] Kwak D, Ha J, Won Y, Kwon Y, Park S (2021). Effects of thyroid-stimulating hormone suppression after thyroidectomy for thyroid cancer on bone mineral density in postmenopausal women: a systematic review and meta-analysis. BMJ Open.

[16] Albayrak T, Yanal H, Sengul D, Sengul I, Albayrak M, Eyüpoğlu S (2023). First management of percutaneous dilatational tracheostomy in severe acute respiratory syndrome coronavirus 2 akin to the vital head and neck region and thyroid gland bed: trust, but be careful whom (you trust)?. Rev Assoc Med Bras (1992).

[17] Tran TV, Kitahara CM, Leenhardt L, Vathaire F, Boutron-Ruault MC, Journy N (2022). The effect of thyroid dysfunction on breast cancer risk: an updated meta-analysis. Endocr Relat Cancer.

[18] Davis PJ, Goglia F, Leonard JL (2016). Nongenomic actions of thyroid hormone. Nat Rev Endocrinol.

[19] Bates JN, Kohn TP, Pastuszak AW (2020). Effect of thyroid hormone derangements on sexual function in men and women. Sex Med Rev.

[20] Sengul D, Sengul I (2018). Are there any variation in neutrophil lymphocyte ratio, mean platelet volume, and platelet count between papillary thyroid cancer and benign nodular thyroid diseases?. Sanamed.

[21] Sengul D, Sengul I (2023). World Thyroid Day 2023 in thyroidology: no overlook thyroid dis-eases to opt for "thyroid health" purposes. Rev Assoc Med Bras (1992).

[22] Sengul I, Sengul D (2023). The 2023 Bethesda system for reporting thyroid cytopathology: novi sub sole, subdivision is no more debatable, in thyroidology. Rev Assoc Med Bras (1992).

[23] Sengul I, Sengul D (2022). May 25-31, International Thyroid Awareness Week & May 25, World Thyroid Day, 2022: ındetermination of indeterminate cytology, AUS/FLUS, FN, SUSP, in thyroidology?. Sanamed.

[24] Sengul I, Sengul D (2024). Evangely, the subcategorization has been announced in the 2023 Bethesda system for reporting thyroid cytopathology: let bygones be bygones in thyroidology!. Rev Assoc Med Bras (1992).

[25] Sengul I, Sengul D (2024). Luminescences of the 2023 Bethesda System for reporting thyroid cytopathology, 3rd edition, in thyroidology. Cerasus J Med.

[26] Samavat H, Kurzer MS (2015). Estrogen metabolism and breast cancer. Cancer Lett.

[27] Yadav M, Kose V, Bhalerao A (2023). Frequency of thyroid disorder in pre- and postmenopausal women and ıts association with menopausal symptoms. Cureus.

